# Advancements in preclinical screening methods for artemisinins during pregnancy

**DOI:** 10.1186/1475-2875-9-S2-P8

**Published:** 2010-10-20

**Authors:** G Donegan, C A Forrester, S A Ward

**Affiliations:** 1 Molecular & Biochemical Parasitology Group, Liverpool School of Tropical Medicine, Liverpool, L3 5QA, UK

## Background

An unprecedented effort from public and private international health agencies has been made to address the urgent issue of malaria in pregnancy.

The selection of antimalarial drug therapy during pregnancy currently depends upon the severity of the disease, gestational age (stage of fetal development) and patterns of drug resistance in the area. To combat multi-drug resistance the WHO recommends include the use of Artemisinin-based combination therapies.

To assist in policy formulation of arteminisin drug combination therapy during pregnancy, artemisinin-derivatives are currently undergoing preÂ¬clinical risk:benefit assessments [1]. Recent animal trials have indicated that arteminisins during pregnancy result in fetal morbidity, low birthweight, cardiac and skeletal birth defects [2].

Currently validated preclinical teratogenicity/embryolethality drug screenÂ¬ing includes whole embryo culture (WEC), Micromass (MM) limb bud and embryonic stem cell (ESC) culture together with *in vivo* studies to assess embryonic development [3,4].

However while *in vivo* screening assays to gestational day (GD) 13+ enable unambiguous assessment of artemisinin-associated embryotoxicity, standard *in vitro* 48hr WEC studies from 9-11 GD end before the teratogenic effects on vulnerable organ systems impacted by the drug can be evaluated. To address this problem, the WEC experiment is extended to 72 hours: after vascular and limb bud development.

## Materials and methods

For *in vivo* and *in vitro* studies timed-mated Sprague Dawley female rats were administered with artesunate at 9 and 10GD (organogenesis). Validated total morphological scoring systems are used to quantify development of the heart, brain, mandibular and maxillary processes, skeletal formation and otic and optic development [2]. Embryonic blood was collected for haematological analysis and *de novo* blood vessel formation of blood vessels in the yolk-sac was also assessed.

## Results

Extended (72 hours from gestational day 9-12) whole embryo culture times clearly showed artesunate (an artemisinin-derivative) to have dose-dependent effects with total morphological scores increasing as dose was reduced. Unequivocal effects were upon embryonic vascularisation, fore-limb bud development, morphological scores and 50% reduced fetal weight were clearly demonstrated that would not have been seen after 48 hours of culture.

**Figure 1:**
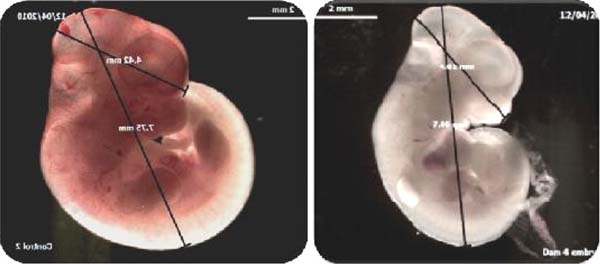
*In vivo* Control (L) and Artesunate-treated (17mg/kg) gestation day 12.

**Figure 2:**
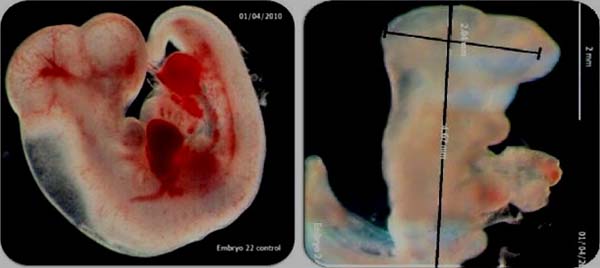
*In vitro* Control (L) and Artesunate-treated (0.5μg/mL) gestation day 12WECs at 72hr.

Analogous effects were confirmed in the *in vivo* experiments at 17mg/kg (Fig 1).The earliest effects were observed in yolk-sac size and vascularisation showing 50% reduction in fetal weight, 20% reduction in head size and severely reduced vascular development at 13GD. There was also a 40% reduction in numbers of nucleated erythroid progenitors. Figure 2.

## Conclusions

These studies seek improve harmonization between the current in vitro developmental toxicity assays and in vivo assays for use in preclinical drug testing. The results indicate that for compounds with teratogenic potential specifically on skeletal and cardiac development, 72hr whole embryo culture assay is a prerequisite for valid *in vitro* assessment and to enable more relevant equivalence with *in vivo* data.
